# Supplementary material for: Stabilization of Acne Vulgaris-Associated Microbial Dysbiosis with 2% Supramolecular Salicylic Acid
Source: Pharmaceuticals (Basel). 2023 Jan 8;16(1):87. doi: 10.3390/ph16010087 (PMC9864713; doi:10.3390/ph16010087)
Supplement: Supplementary file 1 [file pharmaceuticals-16-00087-s001.zip › pharmaceuticals-2090479-supplementary.pdf]

Supplementary Data

Figure S1

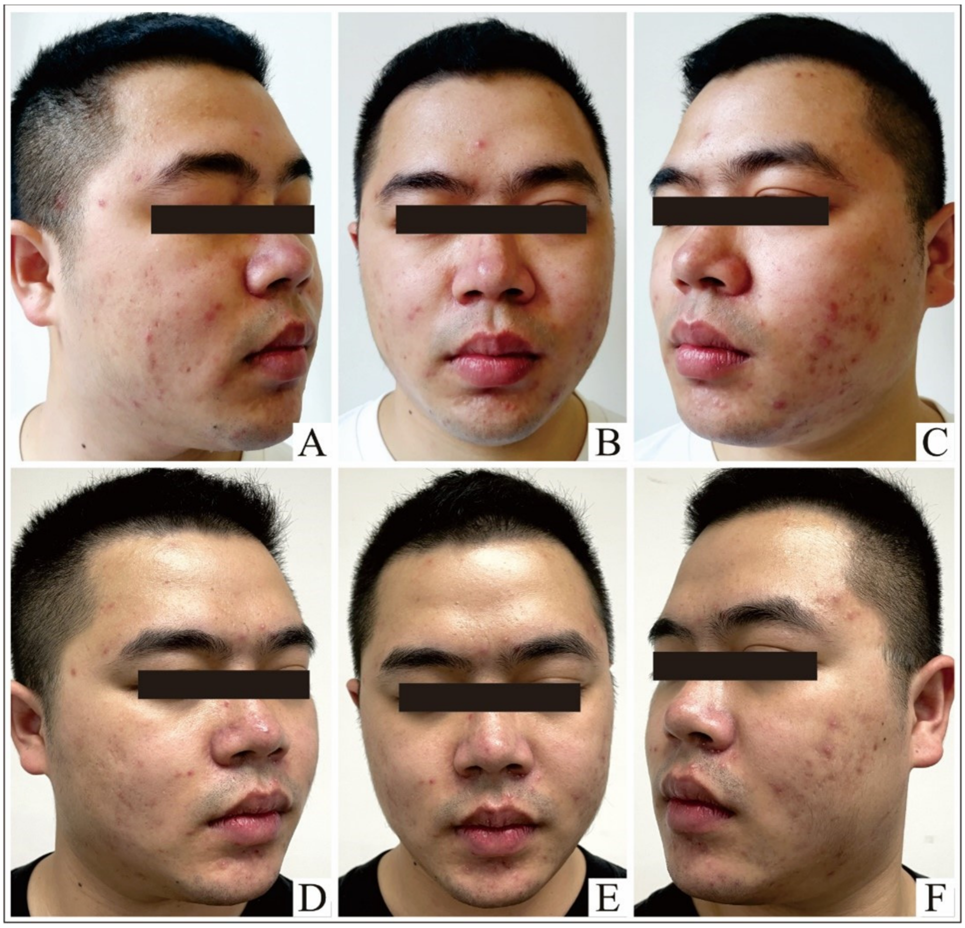

Figure S1 Acne vulgaris patient A, B, and C is before treatment, and D, E, and F is after eight weeks of 2% SSA treatment.

Figure S2:

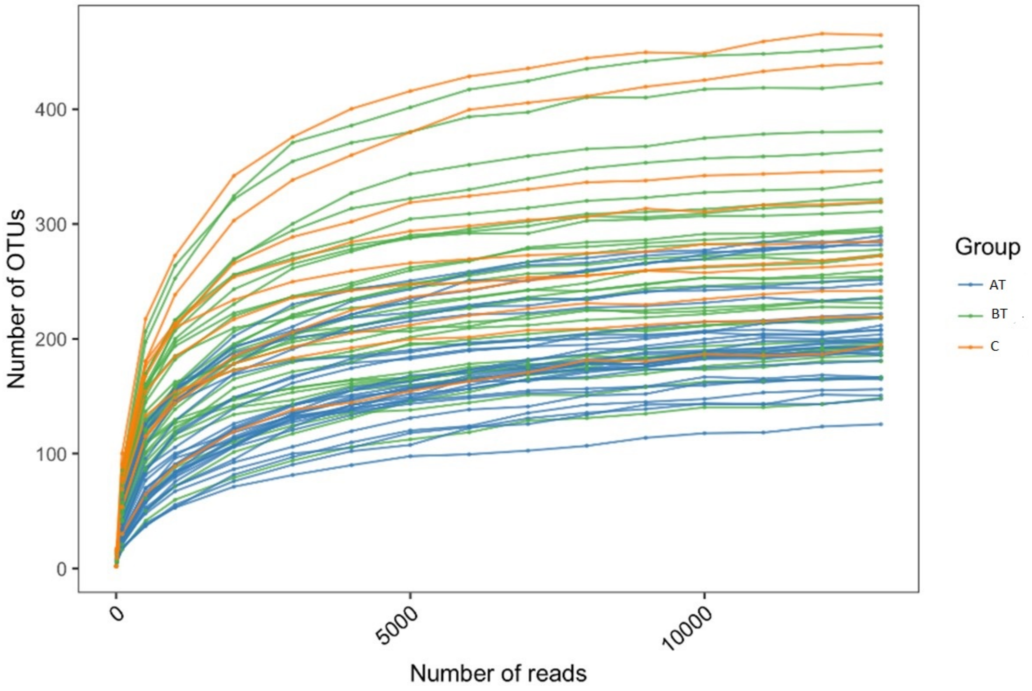

Figure S2 Dilution curve of each sample, BT for before-treated acne samples, AT for after treatment with 2%SSA, and C for control

Figure S3

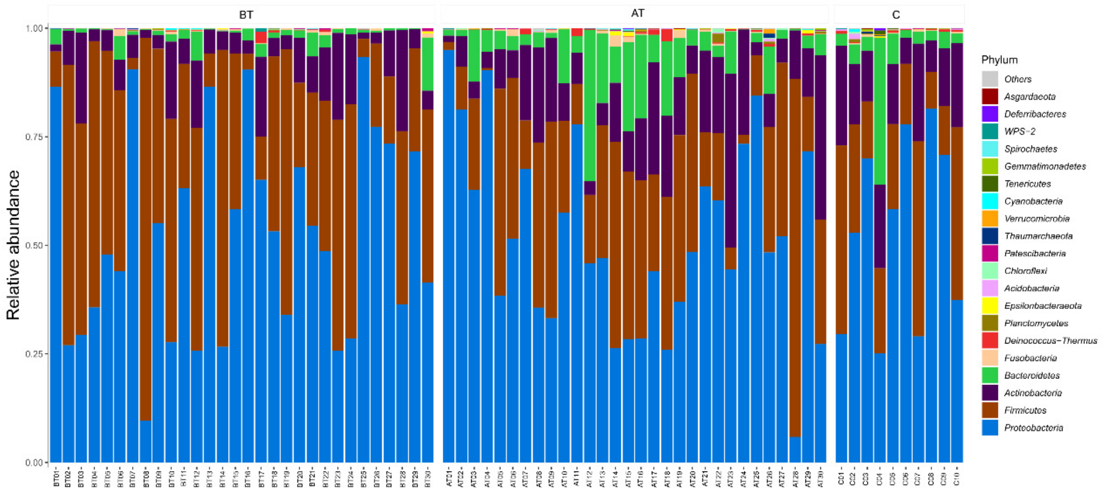

Figure S3 Relative abundance of facial microbiota at phylum level among the pre-treated (BT) (n= 30), treated (AT) (n=30), and (C) (n=10) acne groups, Barplot presenting mean relative abundance in terms of percentage for each sample.

Figure S4

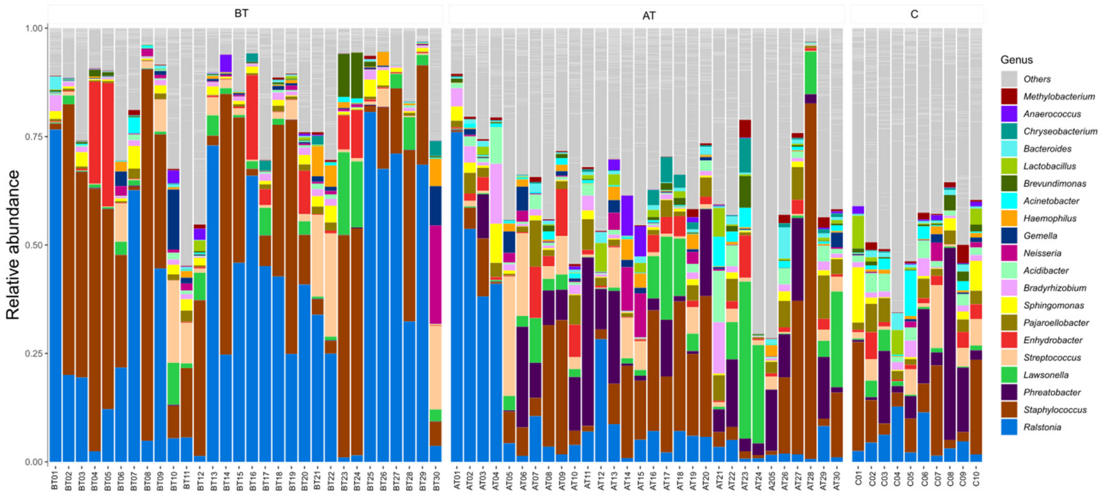

Figure S4 Relative abundance of facial microbiota at genus level among the pre-treated (BT) (n=30), treated (AT) (n=30), and control (C) (n=10) groups. A: Barplot presenting relative abundance in terms of percentage for each sample.

Table S1: Changes in bacterial α-diversity of the pre-treated (BT), post-treatment (AT), and (C) groups in response to 2% SSA treatment.

Groups	Chao1	Shannon	Simpson	Faith's PD
**BT Group**	123.7122 ± 30.6102	1.9807 ± 0.7235	0.6654 ± 0.1718	10.2221 ± 2.3806
**AT Group**	179.2085±64.9888	3.0285 ± 0.7674	0.8406±0.1501	13.4247 ± 3.5314
**C Group**	216.5951 ± 76.4681	3.7105 ± 0.5069	0.9289 ± 0.0530	15.3615 ± 2.6681

Footnotes; BT: Before treatment; AT: After treatment; C group: Control (Healthy)

Table S2: Bacterial composition of the ZymoBIOMICSTM Microbial Community DNA Standard expressed as 16S rRNA gene percentages.

**Bacterial composition**	**Standard***（%）	**Analysis****（%）
Bacillus subtilis	17.4	19.75
Staphylococcus aureus	15.5	14.95
Lactobacillus fermentum	18.4	16.65
Listeria monocytogenes	14.1	13.35
Escherichia coli	10.1	9.2
Salmonella enterica	10.4	9.55
Enterococcus faecalis	9.9	8.95
Pseudomonas aeruginosa	4.2	4.55

Footnote
*Community composition of the standard
**community composition after the actual analysis and testing of the standard.
